# Hitting the floor: Understanding migration patterns following the first episode of psychosis

**DOI:** 10.1016/j.healthplace.2014.04.010

**Published:** 2014-07

**Authors:** James B. Kirkbride

**Affiliations:** Sir Henry Dale Fellow, Division of Psychiatry, University College London, UCL, 2nd Floor Charles Bell House, 67-73 Riding House Street, London W1W 7E, UK

**Keywords:** Social determinants, Social drift, Migration, Schizophrenia, Public mental health

## Abstract

Recent research published in *Health and Place* (Ngamini Ngui et al., 2013b) found that one third of people with first episode psychosis [FEP] will have made a large-scale migration six years after initial diagnosis. Here, I extend this discussion around three important observations. Namely, at first presentation the most disadvantaged communities already shoulder the burden of psychotic morbidity; people with FEP in more rural communities migrate less often, and; people with FEP exhibit both upwards and downwards social mobility after onset. Understanding the reasons for (non-)migration before and after psychosis onset is now required for effective public mental health and service provision.

## 1 Introduction

The paper published by Ngamini Ngui et al. (2013b) in the March 2013 edition of this Journal provides a rare, and very welcome opportunity to understand the residential stability of people in the years following the first episode of psychosis [FEP]. Such research provides important information for mental health service planners in order to allocate resources where they are most likely to be needed.

The findings from this study, based on prospectively collected data in a large cohort of people with FEP (*n*=6873), show that in the first six years following diagnosis over one third of the sample will move to a different health territory. Based on comparative data provided by the authors, this is likely to be in excess of twice the rate for people diagnosed with diabetes, or in the general population, confirming that people with FEP are a highly mobile population (Lix et al., 2006), for whom health and social care services must be provided with sufficient flexibility to ensure smooth transitions in care (McCarthy et al., 2007). Ngamini Ngui et al. (2013b) go on to show that the hazard of migration amongst people with FEP is elevated for men, those aged less than 44 years old, people with a history of migration in the five years preceding FEP and those residing in more socially deprived neighbourhoods at baseline. Interestingly, comorbid diagnoses only increased the hazard of migration in people with FEP residing in rural communities, which Ngamini Ngui et al. (2013b) hypothesise may be due to a greater need for people with complex case histories to find specialized healthcare, typically not provided in more rural regions. Three issues emerge from this study, warranting further comment.

## 2 Baseline disadvantage

First, and perhaps most importantly, Ngamini Ngui et al. (2013b) report that the most disadvantaged neighbourhoods shoulder the greatest burden of psychotic morbidity at baseline. Thus, in people with FEP on whom the authors also had information regarding baseline neighbourhood deprivation (*n*=6354; 92.4% of the cohort), 49.2% and 39.8% were already living in neighbourhoods in the third tertile (highest level) of social and material deprivation, respectively. This pattern accords with research in London (Kirkbride et al., 2008, 2014), which reveals that the incidence of schizophrenia and other non-affective psychotic disorders is greatest in more socioeconomically deprived and unequal communities.

Given difficulties in maintaining employment, income or education during the prodromal phase of disorder (Fusar-Poli et al., 2010; Niendam et al., 2009), it is possible that a proportion of people drift into more disadvantaged communities before FEP, suggesting that early intervention in psychosis efforts should be redoubled in our poorest communities. Such services seek to intervene as soon as possible following the onset of psychotic symptoms, in order to reduce the duration of untreated psychosis [DUP], with longer DUP associated with poorer prognosis, lower quality of life and worse social and functional outcomes (Marshall et al., 2005).

It is likely that social drift only partially explains the preponderance of FEP in our most disadvantaged communities. Other longitudinal cohort studies have demonstrated strong, robust associations between urban birth and upbringing and later schizophrenia risk (Lewis et al., 1992; Mortensen et al., 1999; Zammit et al., 2010), suggesting that cumulative exposure to social or material disadvantage may be aetiologically important. It is likely, therefore, that many of the socioenvironmental determinants of psychosis, as well as the societal burden following onset, are placed upon our poorest communities, who in effect become doubly disadvantaged. This public health tragedy appears to arise irrespective of typically reported national differences in inequality (Wilkinson and Pickett, 2010), as research from Canada (Ngamini Ngui et al., 2013b), Scandinavia (Mortensen et al., 1999; Zammit et al., 2010), the USA (Faris and Dunham, 1939; Silver et al., 2002) and the UK (Kirkbride et al., 2014) illustrate, raising the possibility that disadvantage relative to those in your immediate society may be key drivers, or reservoirs of sustained psychiatric morbidity. We also know that some foreign-born immigrant populations and their descendants, who are often overrepresented in more disadvantaged, urban communities, show elevated risk of psychosis (Bourque et al., 2010; Cantor-Graae and Selten, 2005; Coid et al., 2008), independent of urban living (Kirkbride et al., 2014). There is therefore a strong case for sustained public mental health investment in our most disadvantaged communities, in terms of both strategies to reduce exposure to the deleterious effects of disadvantage, and strategies to ensure adequate resourcing of health and social care services for people with serious mental illness.

## 3 Geographical migration, social mobility

Ngamini Ngui et al. (2013b) studied time to first migration between health territories in Quebec. These are large regions, meaning that on average, the migration involved was also relatively large. The authors speculate that decisions for such moves might be made by people with FEP seeking better mental health care services. There is some support for this hypothesis in the data; of the 295 people with FEP living in rural areas at baseline who moved between health territories over the follow-up period, nearly 76% (*n*=224) migrated to more urban areas, which the authors speculated might be due to help-seeking behaviours. However, this number actually reflects a minority of all people with FEP living in rural areas at baseline (i.e. 224 of 1101; 20.3%); most people with FEP living in rural areas at baseline do not move over the follow-up period (*n*=806; 73.2%). The proportion of people with FEP who do not migrate from small towns in this study was even greater (78.4%). From a public health perspective, the greater concern – in terms of both relative and absolute risk – lies in urban areas; the proportion of non-migrants falls to 61.5% of all people with FEP living in urban areas at baseline, while migrants from urban areas with FEP also represent the largest single migratory group of the entire sample (*n*=1883; 27.4%). This suggests that urban residents with FEP experience the greatest geographical instability and social mobility. This will have implications for the provision of mental health services in urban areas, which must be flexible enough to adapt to provide continuity of care to a relatively mobile population group (McCarthy et al., 2007).

Future research should examine the reasons why people with FEP migrate, as well as reasons why they do not. Older hypotheses of the raised rates in first generation immigrants posited that people in the prodromal phase of schizophrenia may be predisposed to migrate, though empirical research now suggests this is unlikely to explain the excess rates in international migrants (Selten et al., 2002). However, as the work by Ngamni Ngui makes plain, internal (within-country) social and geographical mobility following FEP onset exists, and warrants public health attention. An alternative reading of Ngamini Ngui et al. (2013b) research is that the relative geographical and temporal stability of people with FEP in more rural areas (relative to those in urban areas) suggests that rural areas may offer more stable, socially supportive communities. Indeed, there is strong evidence that increased neighbourhood residential instability decreases social integration and support, by reducing the opportunity to develop effective friendship ties (Sampson, 1988). The relatively large moves studied by Ngamini Ngui et al. (2013b) (i.e. between health territories) may also disrupt social support networks for people with, or following FEP (Silver et al., 2002). A recent study by (Paksarian et al., in press) demonstrated that similarly large, and presumably, disruptive moves amongst children and adolescents in Denmark, were associated with a greater risk of psychotic disorder, and the number of school moves in early childhood appears to be related to psychotic-like symptoms at 12 years old (Singh et al., 2014). Studies are now required which investigate the associations between migration, residential stability, social support and social and functional outcomes in people with FEP (Sibitz et al., 2011). Such effects may be particularly deleterious in, or with migration to more urban environments (Schomerus et al., 2007), which typically have higher levels of social fragmentation as a consequence of greater population turnover and social inequalities.

## 4 Beware of hitting the floor: understanding the limits of social drift

Ngamini Ngui et al. (2013b) argue their data support the theory that people with FEP drift into more socially and materially disadvantaged neighbourhoods. Evidence cited for this from their study includes the fact that over 80% of migrants with FEP living in the most affluent tertile at baseline will move into a more socially disadvantaged health territory during the follow-up period. Expressed as a proportion of all people with FEP in the cohort, 9.1% of the sample migrated to a *more* socially disadvantaged tertile over the follow-up period. However, 8.2% migrated to a *less* socially disadvantaged tertile (i.e. upward social mobility), a net difference of just under one per cent in the direction of downward social drift. Reasons for this are unknown, but returning to the family home following the onset of disorder for health care and social support may provide a possible explanation. When the same analysis is applied to material disadvantage, net downward migration shrinks to 0.4%. But these net flows clearly ignore floor and ceiling effects present in the data. In short, people in the most affluent tertiles of social or material deprivation cannot migrate to a more affluent tertile; the only direction of migration permissible (as the data are categorised) are within tertile, or downwards. This is a ceiling effect. Conversely, people in the most disadvantaged tertiles at baseline cannot migrate or drift down further, since they are already in the most socially or materially deprived communities, as categorised. And because, as we saw earlier, people with FEP are over-represented in the most disadvantaged tertiles at baseline, floor effects will have a greater bearing on the data than ceiling effects, suggesting that the net migration statistics above will underestimate social drift.

If we assume that upward migration (in the least disadvantaged tertile) and downward migration (in the most disadvantaged tertile) were possible (had the data been categorised across more groups), and, crudely, would have occurred at the same overall proportions as empirically observed by Ngamini Ngui et al. (2013b) in other tertiles, an adjustment to net migration flows can be made in the presence of floor and ceiling effects. This is achieved by weighting the proportion of people with FEP in the sample who migrated (upwards or downwards) by the inverse probability that they had the opportunity to move. Thus, weighted net migration flows would have been −7.1% for social disadvantage (towards more disadvantaged areas) and −3.3% for material deprivation, suggestive of modest overall social drift. However 4.9% (*n*=337) of the sample migrated but in an unknown direction. The effect this would have had on net migration, had the direction been known, would have depended on the distribution of missing data across tertiles of disadvantage, and according to the direction of migration. If all those with missing data had migrated upwards, from either of the two most disadvantaged tertiles, weighted net migration for social disadvantage would have been virtually nil (data available from author). Had all people with missing data migrated downwards, from the two least disadvantaged tertiles, weighted net migration would have strongly favoured drift into more socially disadvantaged communities (~14.5%). A similar pattern occurs for material disadvantage.

The work by Ngamini Ngui et al. (2013b) highlights an urgent need to address socioeconomic disparities in the distribution of psychotic disorders in our populations, with more disadvantaged communities harbouring the burden of incidence, prevalence and the long term burden of care and management of the course and outcomes of psychotic disorders. Although people with FEP experience substantial residential mobility after onset, the excess risk of schizophrenia in certain communities, and particularly deprived, urban environments appears to be stable over time (Ngamini Ngui et al., 2013a), underlining the importance of distinguishing between individual- and neighbourhood-level spatio-temporal mobility. These findings highlight the need to promote population-based strategies to prevent exposure to deleterious environmental risk factors for disorder before and after psychosis onset (social and ethnic fragmentation, low social cohesion or support, economic deprivation) (Kirkbride and Jones, 2011). We also need to provide disadvantaged communities with sufficient resources which foster and encourage informal social support and formal mental health care service provision. This need appears strongest in urban populations, where the absolute burden of disorder is greatest, and where the geographical stability of people with FEP may be weakest. The work raises the need for research to link internal migration patterns in people with FEP with the reasons for such migration (or non-migration), and to investigate whether these lead to changes in social, functional and clinical outcomes. Studies should also consider the roles of ethnic minority position and international immigration in the context of residential instability following psychosis onset. While downward social drift operates for a proportion of people with psychosis, this is partially offset by upward social mobility for others; reasons for both need to be better understood.
